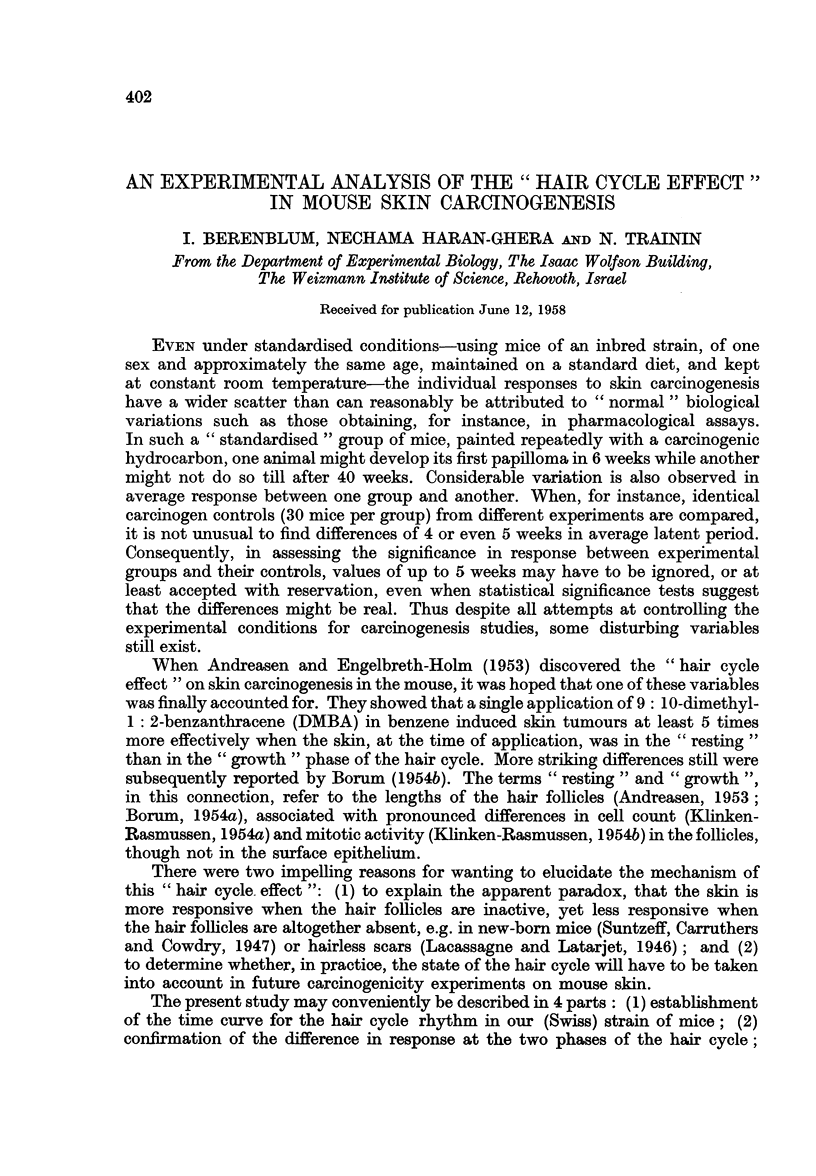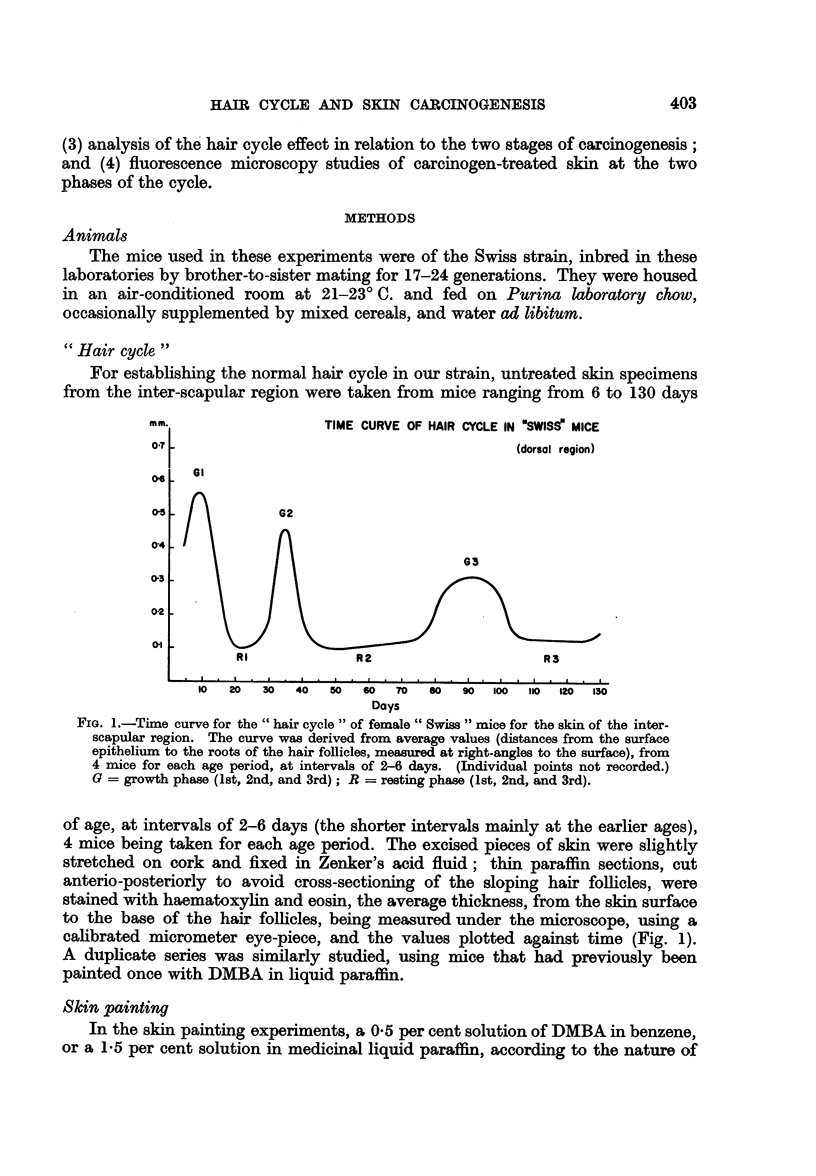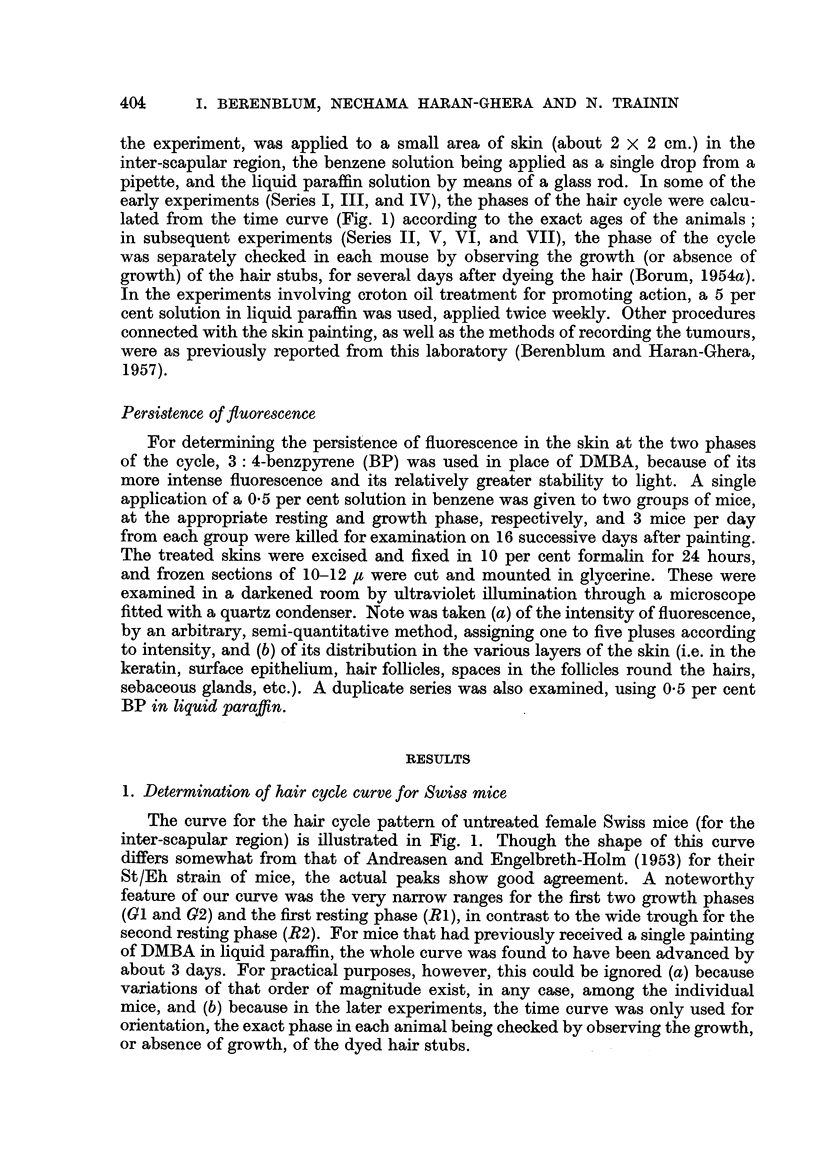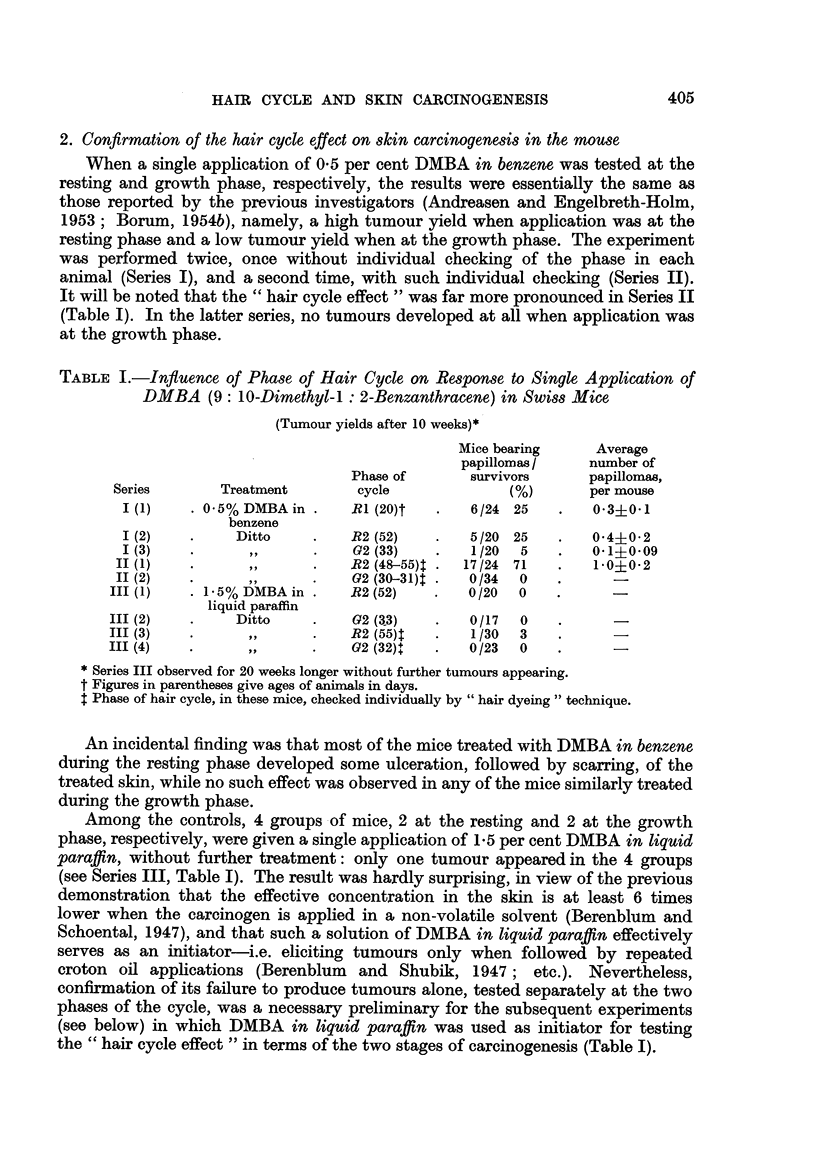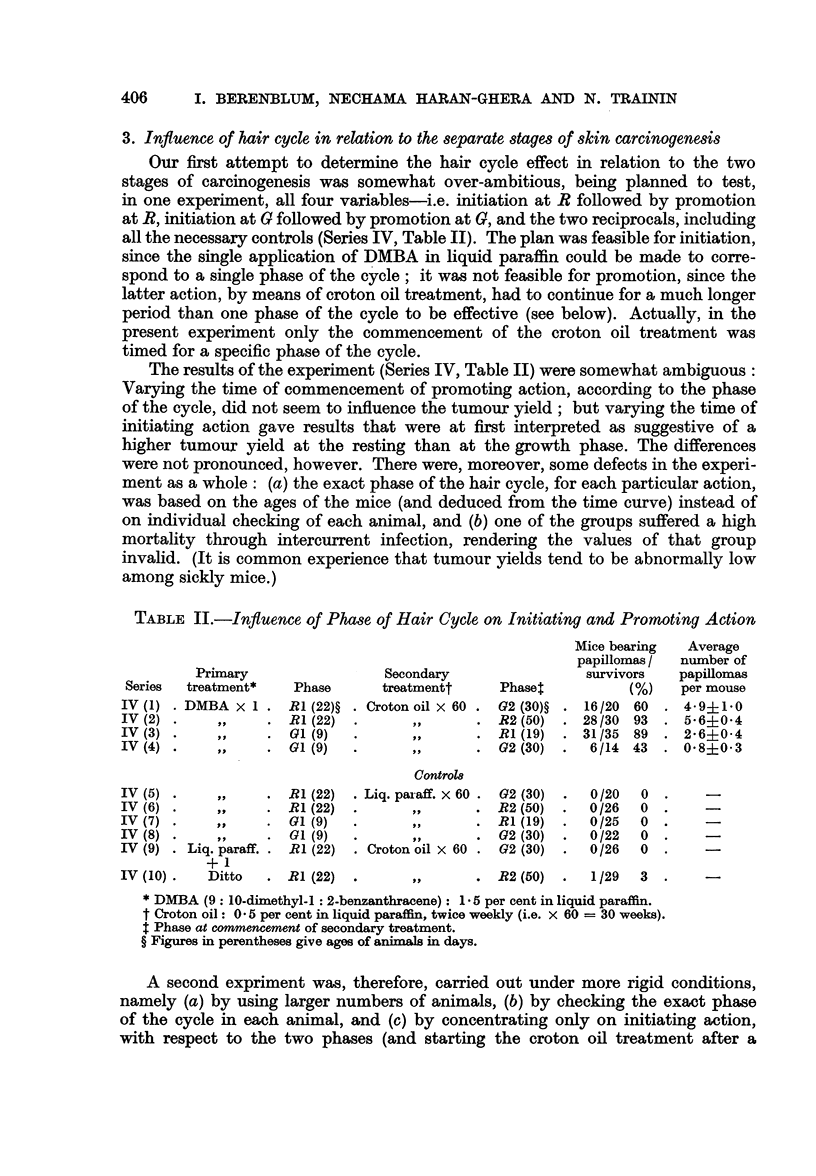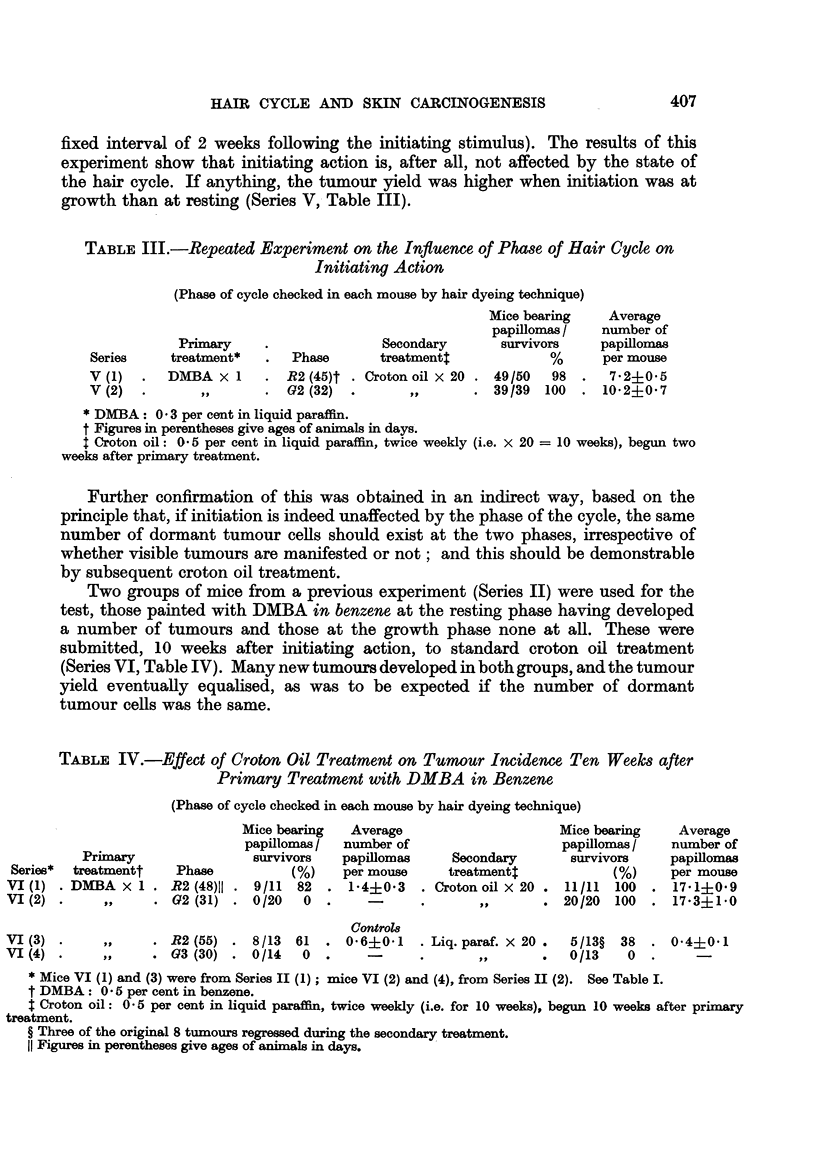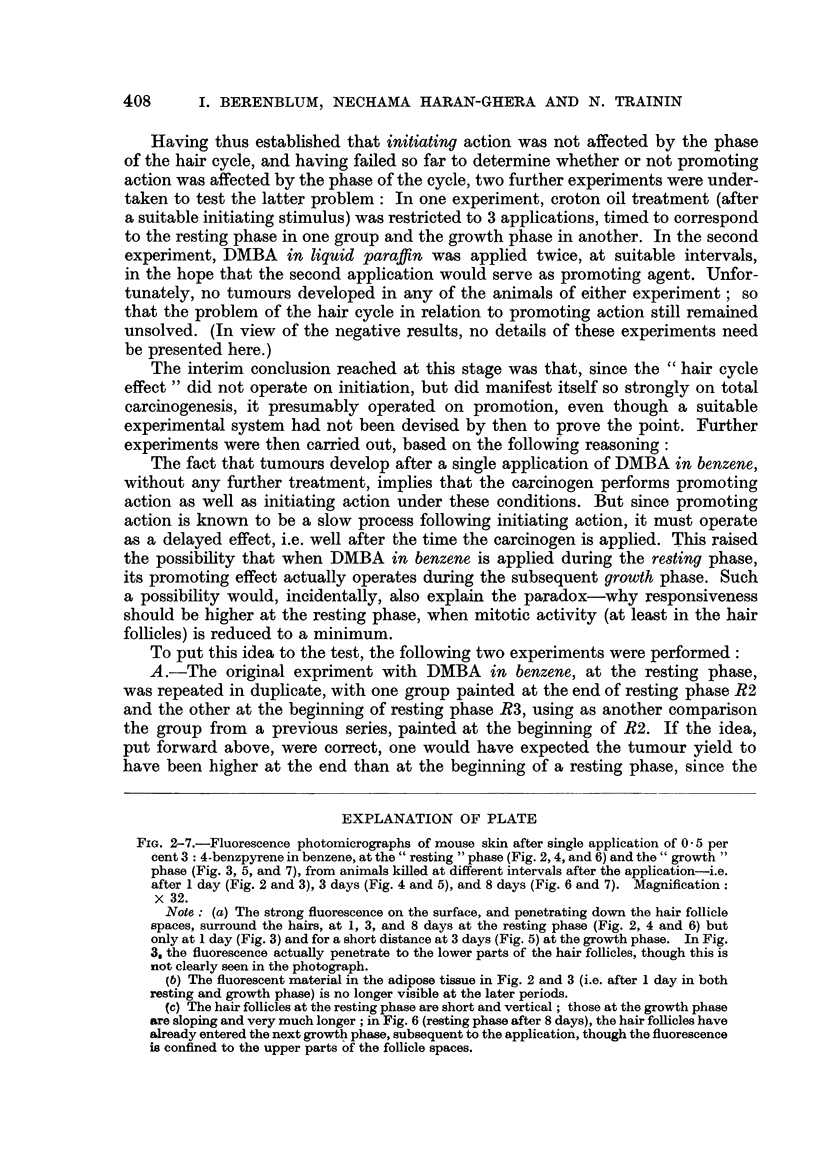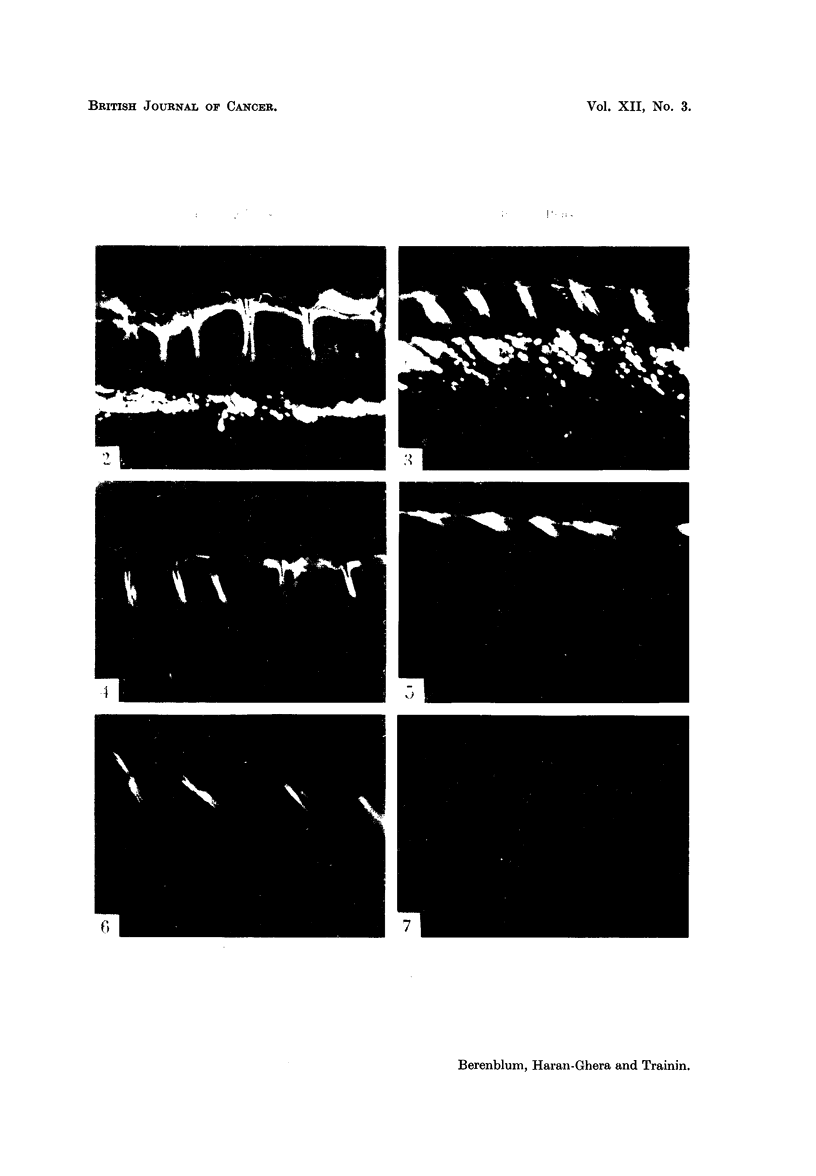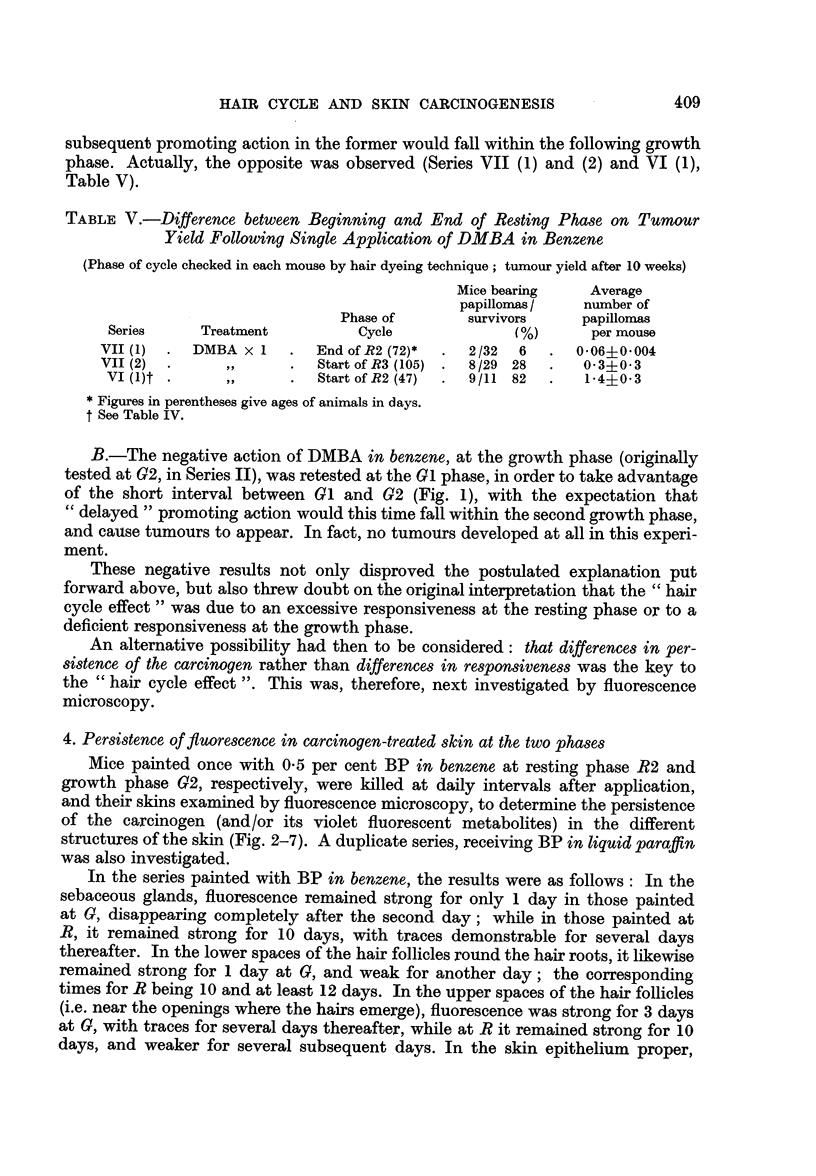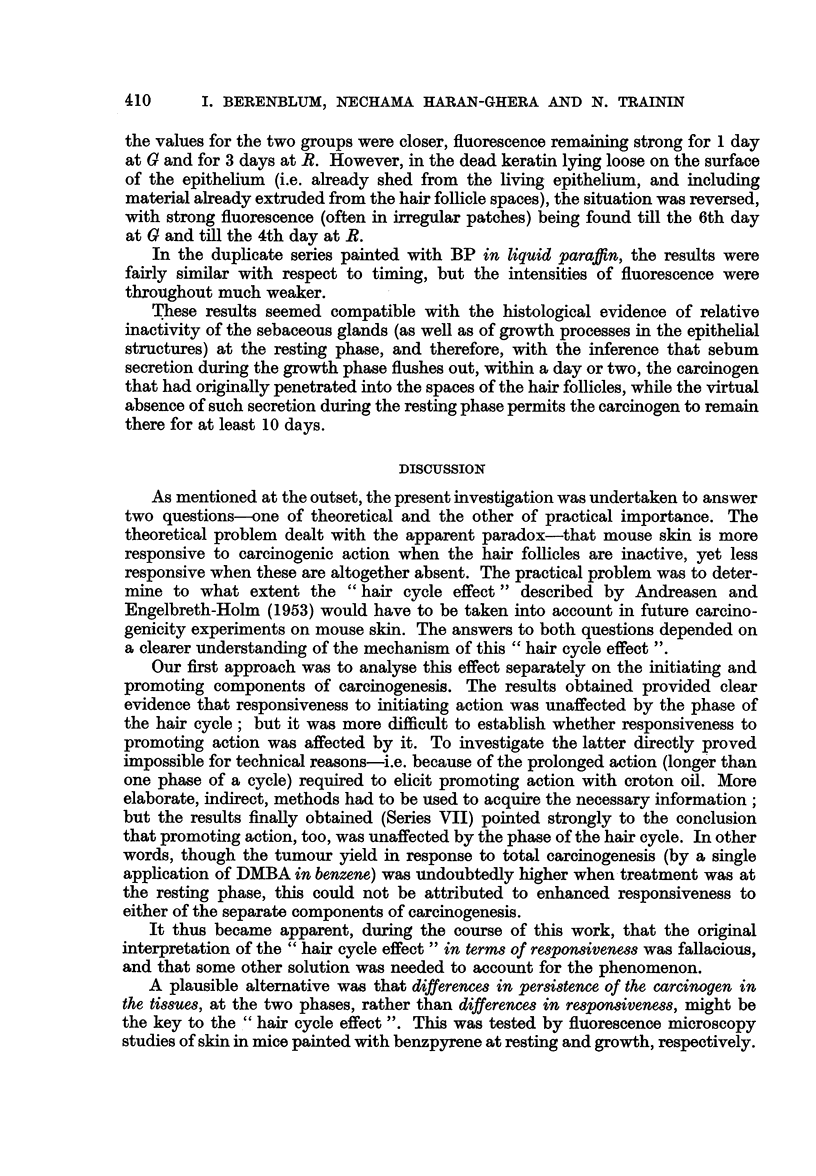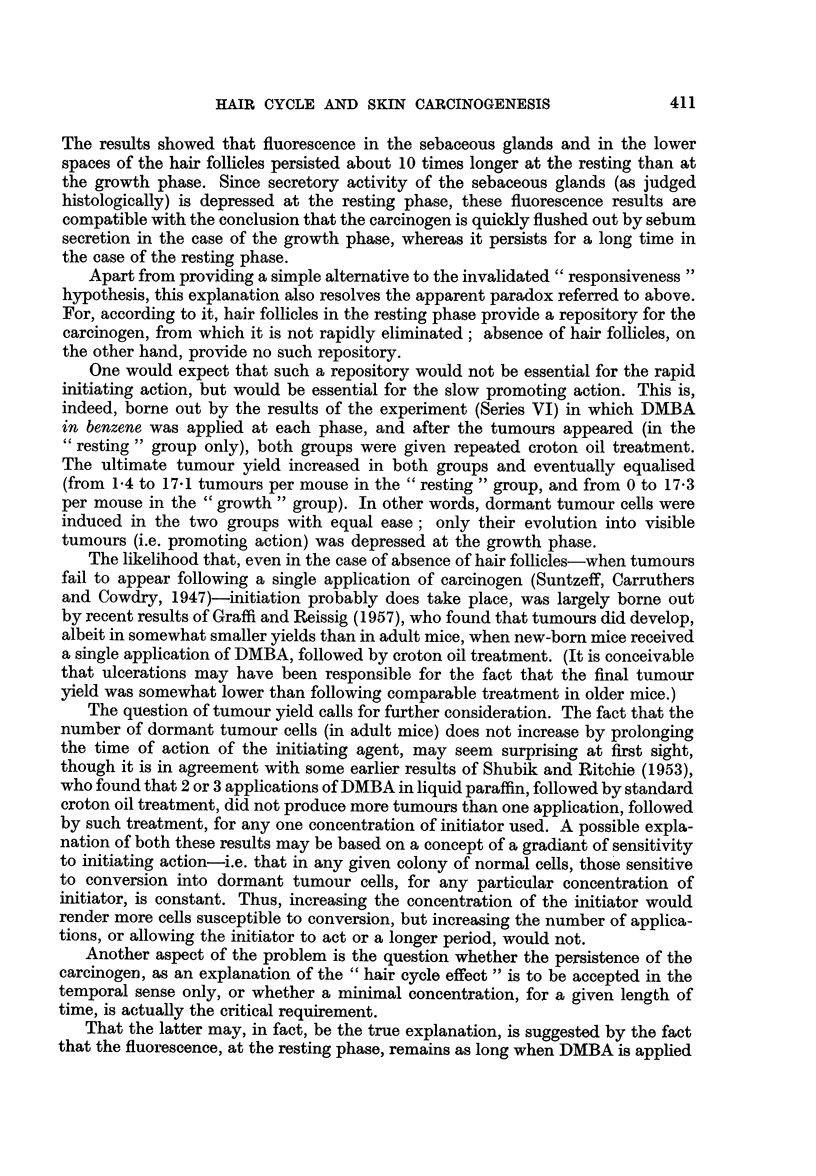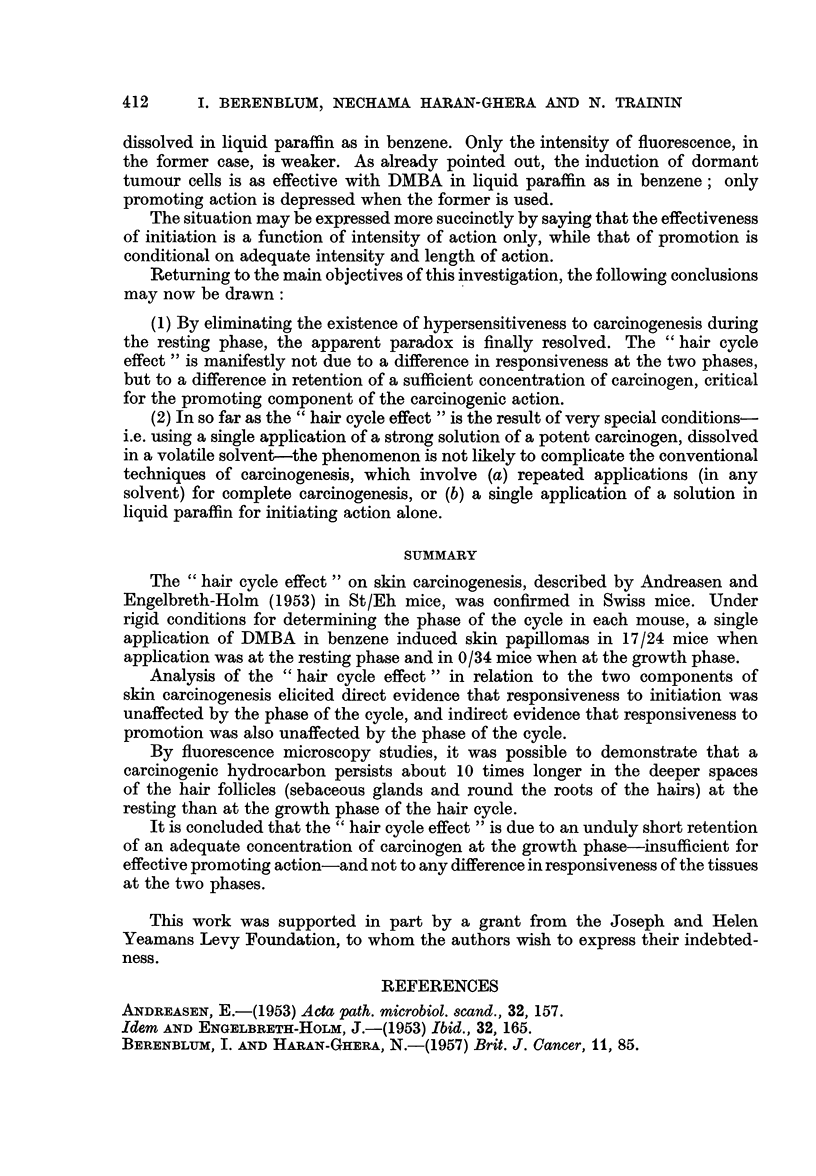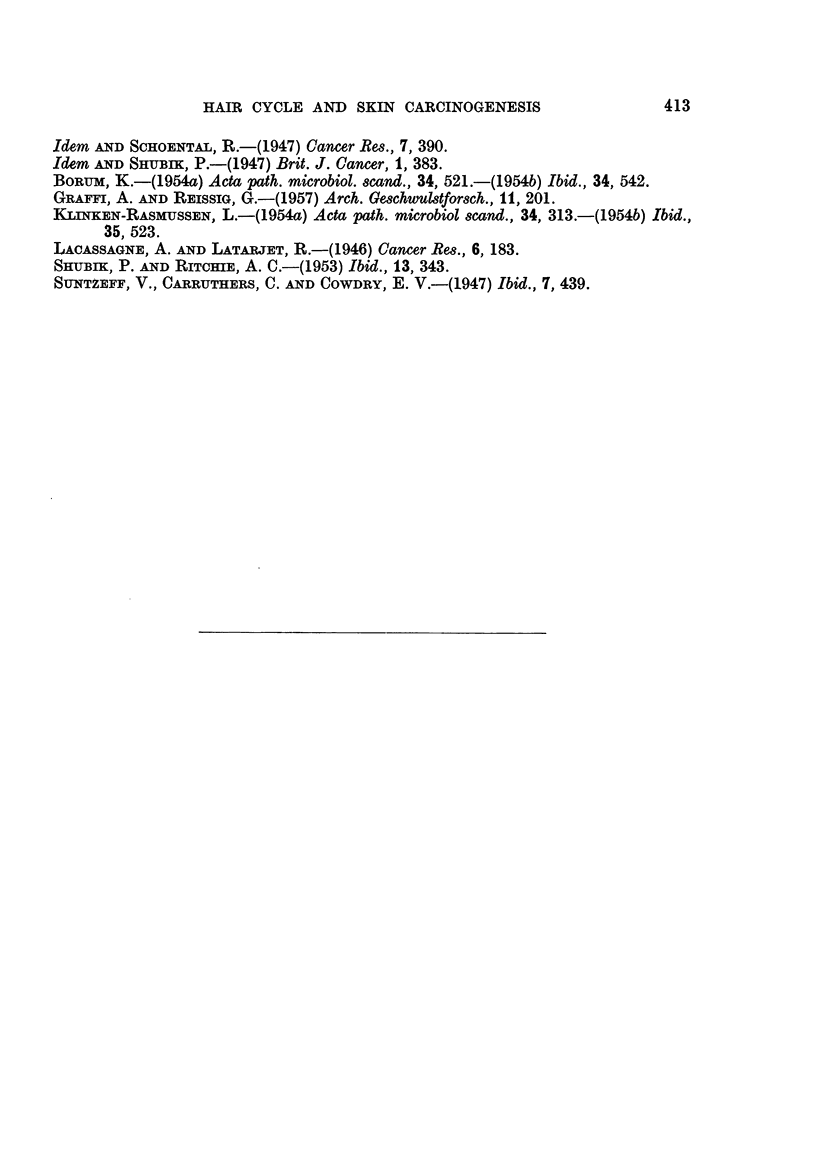# An Experimental Analysis of the “Hair Cycle Effect” in Mouse Skin Carcinogenesis

**DOI:** 10.1038/bjc.1958.48

**Published:** 1958-09

**Authors:** I. Berenblum, Nechama Haran-Ghera, N. Trainin

## Abstract

**Images:**


### AN EXPERIMENTAL ANALYSIS OF THE " HAIR CYCLE EFFECT " IN MOUSE SKIN CARCINOGENESIS

I. BERENBLUM, NECHAMA HARAN-GHERA AND N. TRAININ

*From the Department of Experimental Biology, The Isaac Wolfson Building, The Weizmann Institute of Science, Rehovoth, Israel*

Received for publication June 12, 1958

EVEN under standardised conditions—using mice of an inbred strain, of one sex and approximately the same age, maintained on a standard diet, and kept at constant room temperature—the individual responses to skin carcinogenesis have a wider scatter than can reasonably be attributed to " normal " biological variations such as those obtaining, for instance, in pharmacological assays. In such a " standardised " group of mice, painted repeatedly with a carcinogenic hydrocarbon, one animal might develop its first papilloma in 6 weeks while another might not do so till after 40 weeks. Considerable variation is also observed in average response between one group and another. When, for instance, identical carcinogen controls (30 mice per group) from different experiments are compared, it is not unusual to find differences of 4 or even 5 weeks in average latent period. Consequently, in assessing the significance in response between experimental groups and their controls, values of up to 5 weeks may have to be ignored, or at least accepted with reservation, even when statistical significance tests suggest that the differences might be real. Thus despite all attempts at controlling the experimental conditions for carcinogenesis studies, some disturbing variables still exist.

When Andreasen and Engelbreth-Holm (1953) discovered the " hair cycle effect " on skin carcinogenesis in the mouse, it was hoped that one of these variables was finally accounted for. They showed that a single application of 9 : 10-dimethyl-1 : 2-benzanthracene (DMBA) in benzene induced skin tumours at least 5 times more effectively when the skin, at the time of application, was in the " resting " than in the " growth " phase of the hair cycle. More striking differences still were subsequently reported by Borum (1954*b*). The terms " resting " and " growth ", in this connection, refer to the lengths of the hair follicles (Andreasen, 1953 ; Borum, 1954*a*), associated with pronounced differences in cell count (Klinken-Rasmussen, 1954*a*) and mitotic activity (Klinken-Rasmussen, 1954*b*) in the follicles, though not in the surface epithelium.

There were two impelling reasons for wanting to elucidate the mechanism of this " hair cycle effect ": (1) to explain the apparent paradox, that the skin is more responsive when the hair follicles are inactive, yet less responsive when the hair follicles are altogether absent, e.g. in new-born mice (Suntzeff, Carruthers and Cowdry, 1947) or hairless scars (Lacassagne and Latarjet, 1946) ; and (2) to determine whether, in practice, the state of the hair cycle will have to be taken into account in future carcinogenicity experiments on mouse skin.

The present study may conveniently be described in 4 parts : (1) establishment of the time curve for the hair cycle rhythm in our (Swiss) strain of mice ; (2) confirmation of the difference in response at the two phases of the hair cycle ;

(3) analysis of the hair cycle effect in relation to the two stages of carcinogenesis ; and (4) fluorescence microscopy studies of carcinogen-treated skin at the two phases of the cycle.

### METHODS

### *Animals*

The mice used in these experiments were of the Swiss strain, inbred in these laboratories by brother-to-sister mating for 17–24 generations. They were housed in an air-conditioned room at 21–23° C. and fed on *Purina laboratory chow*, occasionally supplemented by mixed cereals, and water *ad libitum.*

### " *Hair cycle* "

For establishing the normal hair cycle in our strain, untreated skin specimens from the inter-scapular region were taken from mice ranging from 6 to 130 days

FIG. 1.—Time curve for the " hair cycle " of female " Swiss " mice for the skin of the inter-scapular region. The curve was derived from average values (distances from the surface epithelium to the roots of the hair follicles, measured at right-angles to the surface), from 4 mice for each age period, at intervals of 2–6 days. (Individual points not recorded.) $G$ = growth phase (1st, 2nd, and 3rd) ; $R$ = resting phase (1st, 2nd, and 3rd).

of age, at intervals of 2–6 days (the shorter intervals mainly at the earlier ages), 4 mice being taken for each age period. The excised pieces of skin were slightly stretched on cork and fixed in Zenker's acid fluid ; thin paraffin sections, cut anterio-posteriorly to avoid cross-sectioning of the sloping hair follicles, were stained with haematoxylin and eosin, the average thickness, from the skin surface to the base of the hair follicles, being measured under the microscope, using a calibrated micrometer eye-piece, and the values plotted against time (Fig. 1). A duplicate series was similarly studied, using mice that had previously been painted once with DMBA in liquid paraffin.

### *Skin painting*

In the skin painting experiments, a 0·5 per cent solution of DMBA in benzene, or a 1·5 per cent solution in medicinal liquid paraffin, according to the nature of the experiment, was applied to a small area of skin (about 2 × 2 cm.) in the inter-scapular region, the benzene solution being applied as a single drop from a pipette, and the liquid paraffin solution by means of a glass rod. In some of the early experiments (Series I, III, and IV), the phases of the hair cycle were calculated from the time curve (Fig. 1) according to the exact ages of the animals ; in subsequent experiments (Series II, V, VI, and VII), the phase of the cycle was separately checked in each mouse by observing the growth (or absence of growth) of the hair stubs, for several days after dyeing the hair (Borum, 1954*a*). In the experiments involving croton oil treatment for promoting action, a 5 per cent solution in liquid paraffin was used, applied twice weekly. Other procedures connected with the skin painting, as well as the methods of recording the tumours, were as previously reported from this laboratory (Berenblum and Haran-Ghera, 1957).

*Persistence of fluorescence*

For determining the persistence of fluorescence in the skin at the two phases of the cycle, 3 : 4-benzpyrene (BP) was used in place of DMBA, because of its more intense fluorescence and its relatively greater stability to light. A single application of a 0·5 per cent solution in benzene was given to two groups of mice, at the appropriate resting and growth phase, respectively, and 3 mice per day from each group were killed for examination on 16 successive days after painting. The treated skins were excised and fixed in 10 per cent formalin for 24 hours, and frozen sections of 10–12 $\mu$ were cut and mounted in glycerine. These were examined in a darkened room by ultraviolet illumination through a microscope fitted with a quartz condenser. Note was taken (*a*) of the intensity of fluorescence, by an arbitrary, semi-quantitative method, assigning one to five pluses according to intensity, and (*b*) of its distribution in the various layers of the skin (i.e. in the keratin, surface epithelium, hair follicles, spaces in the follicles round the hairs, sebaceous glands, etc.). A duplicate series was also examined, using 0·5 per cent BP *in liquid paraffin.*

### RESULTS

### 1. *Determination of hair cycle curve for Swiss mice*

The curve for the hair cycle pattern of untreated female Swiss mice (for the inter-scapular region) is illustrated in Fig. 1. Though the shape of this curve differs somewhat from that of Andreasen and Engelbreth-Holm (1953) for their St/Eh strain of mice, the actual peaks show good agreement. A noteworthy feature of our curve was the very narrow ranges for the first two growth phases (*G*1 and *G*2) and the first resting phase (*R*1), in contrast to the wide trough for the second resting phase (*R*2). For mice that had previously received a single painting of DMBA in liquid paraffin, the whole curve was found to have been advanced by about 3 days. For practical purposes, however, this could be ignored (*a*) because variations of that order of magnitude exist, in any case, among the individual mice, and (*b*) because in the later experiments, the time curve was only used for orientation, the exact phase in each animal being checked by observing the growth, or absence of growth, of the dyed hair stubs.

2. *Confirmation of the hair cycle effect on skin carcinogenesis in the mouse*

When a single application of 0·5 per cent DMBA *in benzene* was tested at the resting and growth phase, respectively, the results were essentially the same as those reported by the previous investigators (Andreasen and Engelbreth-Holm, 1953; Borum, 1954*b*), namely, a high tumour yield when application was at the resting phase and a low tumour yield when at the growth phase. The experiment was performed twice, once without individual checking of the phase in each animal (Series I), and a second time, with such individual checking (Series II). It will be noted that the "hair cycle effect" was far more pronounced in Series II (Table I). In the latter series, no tumours developed at all when application was at the growth phase.

TABLE I.—*Influence of Phase of Hair Cycle on Response to Single Application of DMBA* (9 : 10-*Dimethyl*-1 : 2-*Benzanthracene*) *in Swiss Mice*

(Tumour yields after 10 weeks)*

| Series | Treatment | Phase of cycle | Mice bearing papillomas / survivors | (%) | Average number of papillomas, per mouse |
|---|---|---|---|---|---|
| I (1) | 0·5% DMBA in benzene | *R*1 (20)† | 6/24 | 25 | 0·3±0·1 |
| I (2) | Ditto | *R*2 (52) | 5/20 | 25 | 0·4±0·2 |
| I (3) | ,, | *G*2 (33) | 1/20 | 5 | 0·1±0·09 |
| II (1) | ,, | *R*2 (48–55)‡ | 17/24 | 71 | 1·0±0·2 |
| II (2) | ,, | *G*2 (30–31)‡ | 0/34 | 0 | — |
| III (1) | 1·5% DMBA in liquid paraffin | *R*2 (52) | 0/20 | 0 | — |
| III (2) | Ditto | *G*2 (33) | 0/17 | 0 | — |
| III (3) | ,, | *R*2 (55)‡ | 1/30 | 3 | — |
| III (4) | ,, | *G*2 (32)‡ | 0/23 | 0 | — |

* Series III observed for 20 weeks longer without further tumours appearing.
† Figures in parentheses give ages of animals in days.
‡ Phase of hair cycle, in these mice, checked individually by "hair dyeing" technique.

An incidental finding was that most of the mice treated with DMBA *in benzene* during the resting phase developed some ulceration, followed by scarring, of the treated skin, while no such effect was observed in any of the mice similarly treated during the growth phase.

Among the controls, 4 groups of mice, 2 at the resting and 2 at the growth phase, respectively, were given a single application of 1·5 per cent DMBA *in liquid paraffin*, without further treatment: only one tumour appeared in the 4 groups (see Series III, Table I). The result was hardly surprising, in view of the previous demonstration that the effective concentration in the skin is at least 6 times lower when the carcinogen is applied in a non-volatile solvent (Berenblum and Schoental, 1947), and that such a solution of DMBA *in liquid paraffin* effectively serves as an initiator—i.e. eliciting tumours only when followed by repeated croton oil applications (Berenblum and Shubik, 1947; etc.). Nevertheless, confirmation of its failure to produce tumours alone, tested separately at the two phases of the cycle, was a necessary preliminary for the subsequent experiments (see below) in which DMBA *in liquid paraffin* was used as initiator for testing the "hair cycle effect" in terms of the two stages of carcinogenesis (Table I).

### 3. *Influence of hair cycle in relation to the separate stages of skin carcinogenesis*

Our first attempt to determine the hair cycle effect in relation to the two stages of carcinogenesis was somewhat over-ambitious, being planned to test, in one experiment, all four variables—i.e. initiation at *R* followed by promotion at *R*, initiation at *G* followed by promotion at *G*, and the two reciprocals, including all the necessary controls (Series IV, Table II). The plan was feasible for initiation, since the single application of DMBA in liquid paraffin could be made to correspond to a single phase of the cycle ; it was not feasible for promotion, since the latter action, by means of croton oil treatment, had to continue for a much longer period than one phase of the cycle to be effective (see below). Actually, in the present experiment only the commencement of the croton oil treatment was timed for a specific phase of the cycle.

The results of the experiment (Series IV, Table II) were somewhat ambiguous : Varying the time of commencement of promoting action, according to the phase of the cycle, did not seem to influence the tumour yield ; but varying the time of initiating action gave results that were at first interpreted as suggestive of a higher tumour yield at the resting than at the growth phase. The differences were not pronounced, however. There were, moreover, some defects in the experiment as a whole : (*a*) the exact phase of the hair cycle, for each particular action, was based on the ages of the mice (and deduced from the time curve) instead of on individual checking of each animal, and (*b*) one of the groups suffered a high mortality through intercurrent infection, rendering the values of that group invalid. (It is common experience that tumour yields tend to be abnormally low among sickly mice.)

TABLE II.—*Influence of Phase of Hair Cycle on Initiating and Promoting Action*

| Series | Primary treatment* | Phase | Secondary treatment† | Phase‡ | Mice bearing papillomas / survivors | (%) | Average number of papillomas per mouse |
|---|---|---|---|---|---|---|---|
| IV (1) | DMBA × 1 | *R*1 (22)§ | Croton oil × 60 | *G*2 (30)§ | 16/20 | 60 | 4·9±1·0 |
| IV (2) | ,, | *R*1 (22) | ,, | *R*2 (50) | 28/30 | 93 | 5·6±0·4 |
| IV (3) | ,, | *G*1 (9) | ,, | *R*1 (19) | 31/35 | 89 | 2·6±0·4 |
| IV (4) | ,, | *G*1 (9) | ,, | *G*2 (30) | 6/14 | 43 | 0·8±0·3 |
| | | | *Controls* | | | | |
| IV (5) | ,, | *R*1 (22) | Liq. paraff. × 60 | *G*2 (30) | 0/20 | 0 | — |
| IV (6) | ,, | *R*1 (22) | ,, | *R*2 (50) | 0/26 | 0 | — |
| IV (7) | ,, | *G*1 (9) | ,, | *R*1 (19) | 0/25 | 0 | — |
| IV (8) | ,, | *G*1 (9) | ,, | *G*2 (30) | 0/22 | 0 | — |
| IV (9) | Liq. paraff. + 1 | *R*1 (22) | Croton oil × 60 | *G*2 (30) | 0/26 | 0 | — |
| IV (10) | Ditto | *R*1 (22) | ,, | *R*2 (50) | 1/29 | 3 | — |

* DMBA (9 : 10-dimethyl-1 : 2-benzanthracene) : 1·5 per cent in liquid paraffin.
† Croton oil : 0·5 per cent in liquid paraffin, twice weekly (i.e. × 60 = 30 weeks).
‡ Phase *at commencement* of secondary treatment.
§ Figures in perentheses give ages of animals in days.

A second expriment was, therefore, carried out under more rigid conditions, namely (*a*) by using larger numbers of animals, (*b*) by checking the exact phase of the cycle in each animal, and (*c*) by concentrating only on initiating action, with respect to the two phases (and starting the croton oil treatment after a fixed interval of 2 weeks following the initiating stimulus). The results of this experiment show that initiating action is, after all, not affected by the state of the hair cycle. If anything, the tumour yield was higher when initiation was at growth than at resting (Series V, Table III).

TABLE III.—*Repeated Experiment on the Influence of Phase of Hair Cycle on Initiating Action*

(Phase of cycle checked in each mouse by hair dyeing technique)

| Series | Primary treatment* | Phase | Secondary treatment‡ | Mice bearing papillomas/ survivors | % | Average number of papillomas per mouse |
|---|---|---|---|---|---|---|
| V (1) | DMBA × 1 | *R*2 (45)† | Croton oil × 20 | 49/50 | 98 | 7·2±0·5 |
| V (2) | ,, | *G*2 (32) | ,, | 39/39 | 100 | 10·2±0·7 |

* DMBA: 0·3 per cent in liquid paraffin.
† Figures in perentheses give ages of animals in days.
‡ Croton oil: 0·5 per cent in liquid paraffin, twice weekly (i.e. × 20 = 10 weeks), begun two weeks after primary treatment.

Further confirmation of this was obtained in an indirect way, based on the principle that, if initiation is indeed unaffected by the phase of the cycle, the same number of dormant tumour cells should exist at the two phases, irrespective of whether visible tumours are manifested or not; and this should be demonstrable by subsequent croton oil treatment.

Two groups of mice from a previous experiment (Series II) were used for the test, those painted with DMBA *in benzene* at the resting phase having developed a number of tumours and those at the growth phase none at all. These were submitted, 10 weeks after initiating action, to standard croton oil treatment (Series VI, Table IV). Many new tumours developed in both groups, and the tumour yield eventually equalised, as was to be expected if the number of dormant tumour cells was the same.

TABLE IV.—*Effect of Croton Oil Treatment on Tumour Incidence Ten Weeks after Primary Treatment with DMBA in Benzene*

(Phase of cycle checked in each mouse by hair dyeing technique)

| Series* | Primary treatment† | Phase | Mice bearing papillomas/ survivors | (%) | Average number of papillomas per mouse | Secondary treatment‡ | Mice bearing papillomas/ survivors | (%) | Average number of papillomas per mouse |
|---|---|---|---|---|---|---|---|---|---|
| VI (1) | DMBA × 1 | *R*2 (48)‖ | 9/11 | 82 | 1·4±0·3 | Croton oil × 20 | 11/11 | 100 | 17·1±0·9 |
| VI (2) | ,, | *G*2 (31) | 0/20 | 0 | — | ,, | 20/20 | 100 | 17·3±1·0 |
| | | | | | *Controls* | | | | |
| VI (3) | ,, | *R*2 (55) | 8/13 | 61 | 0·6±0·1 | Liq. paraf. × 20 | 5/13§ | 38 | 0·4±0·1 |
| VI (4) | ,, | *G*3 (30) | 0/14 | 0 | — | ,, | 0/13 | 0 | — |

* Mice VI (1) and (3) were from Series II (1); mice VI (2) and (4), from Series II (2). See Table I.
† DMBA: 0·5 per cent in benzene.
‡ Croton oil: 0·5 per cent in liquid paraffin, twice weekly (i.e. for 10 weeks), begun 10 weeks after primary treatment.
§ Three of the original 8 tumours regressed during the secondary treatment.
‖ Figures in perentheses give ages of animals in days.

Having thus established that *initiating* action was not affected by the phase of the hair cycle, and having failed so far to determine whether or not promoting action was affected by the phase of the cycle, two further experiments were undertaken to test the latter problem : In one experiment, croton oil treatment (after a suitable initiating stimulus) was restricted to 3 applications, timed to correspond to the resting phase in one group and the growth phase in another. In the second experiment, DMBA *in liquid paraffin* was applied twice, at suitable intervals, in the hope that the second application would serve as promoting agent. Unfortunately, no tumours developed in any of the animals of either experiment ; so that the problem of the hair cycle in relation to promoting action still remained unsolved. (In view of the negative results, no details of these experiments need be presented here.)

The interim conclusion reached at this stage was that, since the " hair cycle effect " did not operate on initiation, but did manifest itself so strongly on total carcinogenesis, it presumably operated on promotion, even though a suitable experimental system had not been devised by then to prove the point. Further experiments were then carried out, based on the following reasoning :

The fact that tumours develop after a single application of DMBA *in benzene*, without any further treatment, implies that the carcinogen performs promoting action as well as initiating action under these conditions. But since promoting action is known to be a slow process following initiating action, it must operate as a delayed effect, i.e. well after the time the carcinogen is applied. This raised the possibility that when DMBA *in benzene* is applied during the *resting* phase, its promoting effect actually operates during the subsequent *growth* phase. Such a possibility would, incidentally, also explain the paradox—why responsiveness should be higher at the resting phase, when mitotic activity (at least in the hair follicles) is reduced to a minimum.

To put this idea to the test, the following two experiments were performed :

*A*.—The original expriment with DMBA *in benzene*, at the resting phase, was repeated in duplicate, with one group painted at the end of resting phase *R*2 and the other at the beginning of resting phase *R*3, using as another comparison the group from a previous series, painted at the beginning of *R*2. If the idea, put forward above, were correct, one would have expected the tumour yield to have been higher at the end than at the beginning of a resting phase, since the

---

EXPLANATION OF PLATE

FIG. 2–7.—Fluorescence photomicrographs of mouse skin after single application of 0·5 per cent 3 : 4-benzpyrene in benzene, at the " resting " phase (Fig. 2, 4, and 6) and the " growth " phase (Fig. 3, 5, and 7), from animals killed at different intervals after the application—i.e. after 1 day (Fig. 2 and 3), 3 days (Fig. 4 and 5), and 8 days (Fig. 6 and 7). Magnification : × 32.

*Note :* (*a*) The strong fluorescence on the surface, and penetrating down the hair follicle spaces, surround the hairs, at 1, 3, and 8 days at the resting phase (Fig. 2, 4 and 6) but only at 1 day (Fig. 3) and for a short distance at 3 days (Fig. 5) at the growth phase. In Fig. 3, the fluorescence actually penetrate to the lower parts of the hair follicles, though this is not clearly seen in the photograph.

(*b*) The fluorescent material in the adipose tissue in Fig. 2 and 3 (i.e. after 1 day in both resting and growth phase) is no longer visible at the later periods.

(*c*) The hair follicles at the resting phase are short and vertical ; those at the growth phase are sloping and very much longer ; in Fig. 6 (resting phase after 8 days), the hair follicles have already entered the next growth phase, subsequent to the application, though the fluorescence is confined to the upper parts of the follicle spaces.

Resting Phase

Growth Phase

2

3

4

5

6

7

subsequent promoting action in the former would fall within the following growth phase. Actually, the opposite was observed (Series VII (1) and (2) and VI (1), Table V).

TABLE V.—*Difference between Beginning and End of Resting Phase on Tumour Yield Following Single Application of DMBA in Benzene*

(Phase of cycle checked in each mouse by hair dyeing technique ; tumour yield after 10 weeks)

| Series | Treatment | Phase of Cycle | Mice bearing papillomas/survivors | (%) | Average number of papillomas per mouse |
|---|---|---|---|---|---|
| VII (1) | DMBA × 1 | End of *R*2 (72)* | 2/32 | 6 | 0·06±0·004 |
| VII (2) | ,, | Start of *R*3 (105) | 8/29 | 28 | 0·3±0·3 |
| VI (1)† | ,, | Start of *R*2 (47) | 9/11 | 82 | 1·4±0·3 |

* Figures in perentheses give ages of animals in days.
† See Table IV.

*B.*—The negative action of DMBA *in benzene*, at the growth phase (originally tested at *G*2, in Series II), was retested at the *G*1 phase, in order to take advantage of the short interval between *G*1 and *G*2 (Fig. 1), with the expectation that " delayed " promoting action would this time fall within the second growth phase, and cause tumours to appear. In fact, no tumours developed at all in this experiment.

These negative results not only disproved the postulated explanation put forward above, but also threw doubt on the original interpretation that the " hair cycle effect " was due to an excessive responsiveness at the resting phase or to a deficient responsiveness at the growth phase.

An alternative possibility had then to be considered : *that differences in persistence of the carcinogen* rather than *differences in responsiveness* was the key to the " hair cycle effect ". This was, therefore, next investigated by fluorescence microscopy.

4. *Persistence of fluorescence in carcinogen-treated skin at the two phases*

Mice painted once with 0·5 per cent BP *in benzene* at resting phase *R*2 and growth phase *G*2, respectively, were killed at daily intervals after application, and their skins examined by fluorescence microscopy, to determine the persistence of the carcinogen (and/or its violet fluorescent metabolites) in the different structures of the skin (Fig. 2–7). A duplicate series, receiving BP *in liquid paraffin* was also investigated.

In the series painted with BP *in benzene*, the results were as follows : In the sebaceous glands, fluorescence remained strong for only 1 day in those painted at *G*, disappearing completely after the second day ; while in those painted at *R*, it remained strong for 10 days, with traces demonstrable for several days thereafter. In the lower spaces of the hair follicles round the hair roots, it likewise remained strong for 1 day at *G*, and weak for another day ; the corresponding times for *R* being 10 and at least 12 days. In the upper spaces of the hair follicles (i.e. near the openings where the hairs emerge), fluorescence was strong for 3 days at *G*, with traces for several days thereafter, while at *R* it remained strong for 10 days, and weaker for several subsequent days. In the skin epithelium proper, the values for the two groups were closer, fluorescence remaining strong for 1 day at *G* and for 3 days at *R*. However, in the dead keratin lying loose on the surface of the epithelium (i.e. already shed from the living epithelium, and including material already extruded from the hair follicle spaces), the situation was reversed, with strong fluorescence (often in irregular patches) being found till the 6th day at *G* and till the 4th day at *R*.

In the duplicate series painted with BP *in liquid paraffin*, the results were fairly similar with respect to timing, but the intensities of fluorescence were throughout much weaker.

These results seemed compatible with the histological evidence of relative inactivity of the sebaceous glands (as well as of growth processes in the epithelial structures) at the resting phase, and therefore, with the inference that sebum secretion during the growth phase flushes out, within a day or two, the carcinogen that had originally penetrated into the spaces of the hair follicles, while the virtual absence of such secretion during the resting phase permits the carcinogen to remain there for at least 10 days.

### DISCUSSION

As mentioned at the outset, the present investigation was undertaken to answer two questions—one of theoretical and the other of practical importance. The theoretical problem dealt with the apparent paradox—that mouse skin is more responsive to carcinogenic action when the hair follicles are inactive, yet less responsive when these are altogether absent. The practical problem was to determine to what extent the " hair cycle effect " described by Andreasen and Engelbreth-Holm (1953) would have to be taken into account in future carcinogenicity experiments on mouse skin. The answers to both questions depended on a clearer understanding of the mechanism of this " hair cycle effect ".

Our first approach was to analyse this effect separately on the initiating and promoting components of carcinogenesis. The results obtained provided clear evidence that responsiveness to initiating action was unaffected by the phase of the hair cycle ; but it was more difficult to establish whether responsiveness to promoting action was affected by it. To investigate the latter directly proved impossible for technical reasons—i.e. because of the prolonged action (longer than one phase of a cycle) required to elicit promoting action with croton oil. More elaborate, indirect, methods had to be used to acquire the necessary information ; but the results finally obtained (Series VII) pointed strongly to the conclusion that promoting action, too, was unaffected by the phase of the hair cycle. In other words, though the tumour yield in response to total carcinogenesis (by a single application of DMBA *in benzene*) was undoubtedly higher when treatment was at the resting phase, this could not be attributed to enhanced responsiveness to either of the separate components of carcinogenesis.

It thus became apparent, during the course of this work, that the original interpretation of the " hair cycle effect " *in terms of responsiveness* was fallacious, and that some other solution was needed to account for the phenomenon.

A plausible alternative was that *differences in persistence of the carcinogen in the tissues*, at the two phases, rather than *differences in responsiveness*, might be the key to the " hair cycle effect ". This was tested by fluorescence microscopy studies of skin in mice painted with benzpyrene at resting and growth, respectively.

The results showed that fluorescence in the sebaceous glands and in the lower spaces of the hair follicles persisted about 10 times longer at the resting than at the growth phase. Since secretory activity of the sebaceous glands (as judged histologically) is depressed at the resting phase, these fluorescence results are compatible with the conclusion that the carcinogen is quickly flushed out by sebum secretion in the case of the growth phase, whereas it persists for a long time in the case of the resting phase.

Apart from providing a simple alternative to the invalidated " responsiveness " hypothesis, this explanation also resolves the apparent paradox referred to above. For, according to it, hair follicles in the resting phase provide a repository for the carcinogen, from which it is not rapidly eliminated ; absence of hair follicles, on the other hand, provide no such repository.

One would expect that such a repository would not be essential for the rapid initiating action, but would be essential for the slow promoting action. This is, indeed, borne out by the results of the experiment (Series VI) in which DMBA *in benzene* was applied at each phase, and after the tumours appeared (in the " resting " group only), both groups were given repeated croton oil treatment. The ultimate tumour yield increased in both groups and eventually equalised (from 1·4 to 17·1 tumours per mouse in the " resting " group, and from 0 to 17·3 per mouse in the " growth " group). In other words, dormant tumour cells were induced in the two groups with equal ease ; only their evolution into visible tumours (i.e. promoting action) was depressed at the growth phase.

The likelihood that, even in the case of absence of hair follicles—when tumours fail to appear following a single application of carcinogen (Suntzeff, Carruthers and Cowdry, 1947)—initiation probably does take place, was largely borne out by recent results of Graffi and Reissig (1957), who found that tumours did develop, albeit in somewhat smaller yields than in adult mice, when new-born mice received a single application of DMBA, followed by croton oil treatment. (It is conceivable that ulcerations may have been responsible for the fact that the final tumour yield was somewhat lower than following comparable treatment in older mice.)

The question of tumour yield calls for further consideration. The fact that the number of dormant tumour cells (in adult mice) does not increase by prolonging the time of action of the initiating agent, may seem surprising at first sight, though it is in agreement with some earlier results of Shubik and Ritchie (1953), who found that 2 or 3 applications of DMBA in liquid paraffin, followed by standard croton oil treatment, did not produce more tumours than one application, followed by such treatment, for any one concentration of initiator used. A possible explanation of both these results may be based on a concept of a gradiant of sensitivity to initiating action—i.e. that in any given colony of normal cells, those sensitive to conversion into dormant tumour cells, for any particular concentration of initiator, is constant. Thus, increasing the concentration of the initiator would render more cells susceptible to conversion, but increasing the number of applications, or allowing the initiator to act or a longer period, would not.

Another aspect of the problem is the question whether the persistence of the carcinogen, as an explanation of the " hair cycle effect " is to be accepted in the temporal sense only, or whether a minimal concentration, for a given length of time, is actually the critical requirement.

That the latter may, in fact, be the true explanation, is suggested by the fact that the fluorescence, at the resting phase, remains as long when DMBA is applied dissolved in liquid paraffin as in benzene. Only the intensity of fluorescence, in the former case, is weaker. As already pointed out, the induction of dormant tumour cells is as effective with DMBA in liquid paraffin as in benzene; only promoting action is depressed when the former is used.

The situation may be expressed more succinctly by saying that the effectiveness of initiation is a function of intensity of action only, while that of promotion is conditional on adequate intensity and length of action.

Returning to the main objectives of this investigation, the following conclusions may now be drawn :

(1) By eliminating the existence of hypersensitiveness to carcinogenesis during the resting phase, the apparent paradox is finally resolved. The "hair cycle effect" is manifestly not due to a difference in responsiveness at the two phases, but to a difference in retention of a sufficient concentration of carcinogen, critical for the promoting component of the carcinogenic action.

(2) In so far as the "hair cycle effect" is the result of very special conditions—i.e. using a single application of a strong solution of a potent carcinogen, dissolved in a volatile solvent—the phenomenon is not likely to complicate the conventional techniques of carcinogenesis, which involve (*a*) repeated applications (in any solvent) for complete carcinogenesis, or (*b*) a single application of a solution in liquid paraffin for initiating action alone.

### SUMMARY

The "hair cycle effect" on skin carcinogenesis, described by Andreasen and Engelbreth-Holm (1953) in St/Eh mice, was confirmed in Swiss mice. Under rigid conditions for determining the phase of the cycle in each mouse, a single application of DMBA in benzene induced skin papillomas in 17/24 mice when application was at the resting phase and in 0/34 mice when at the growth phase.

Analysis of the "hair cycle effect" in relation to the two components of skin carcinogenesis elicited direct evidence that responsiveness to initiation was unaffected by the phase of the cycle, and indirect evidence that responsiveness to promotion was also unaffected by the phase of the cycle.

By fluorescence microscopy studies, it was possible to demonstrate that a carcinogenic hydrocarbon persists about 10 times longer in the deeper spaces of the hair follicles (sebaceous glands and round the roots of the hairs) at the resting than at the growth phase of the hair cycle.

It is concluded that the "hair cycle effect" is due to an unduly short retention of an adequate concentration of carcinogen at the growth phase—insufficient for effective promoting action—and not to any difference in responsiveness of the tissues at the two phases.

This work was supported in part by a grant from the Joseph and Helen Yeamans Levy Foundation, to whom the authors wish to express their indebtedness.

### REFERENCES

ANDREASEN, E.—(1953) *Acta path. microbiol. scand.*, **32**, 157.
*Idem* AND ENGELBRETH-HOLM, J.—(1953) *Ibid.*, **32**, 165.
BERENBLUM, I. AND HARAN-GHERA, N.—(1957) *Brit. J. Cancer*, **11**, 85.

*Idem* AND SCHOENTAL, R.—(1947) *Cancer Res.*, **7**, 390.
*Idem* AND SHUBIK, P.—(1947) *Brit. J. Cancer*, **1**, 383.
BORUM, K.—(1954*a*) *Acta path. microbiol. scand.*, **34**, 521.—(1954*b*) *Ibid.*, **34**, 542.
GRAFFI, A. AND REISSIG, G.—(1957) *Arch. Geschwulstforsch.*, **11**, 201.
KLINKEN-RASMUSSEN, L.—(1954*a*) *Acta path. microbiol scand.*, **34**, 313.—(1954*b*) *Ibid.*, **35**, 523.
LACASSAGNE, A. AND LATARJET, R.—(1946) *Cancer Res.*, **6**, 183.
SHUBIK, P. AND RITCHIE, A. C.—(1953) *Ibid.*, **13**, 343.
SUNTZEFF, V., CARRUTHERS, C. AND COWDRY, E. V.—(1947) *Ibid.*, **7**, 439.